# *Yucca*-derived synthesis of gold nanomaterial and their catalytic potential

**DOI:** 10.1186/1556-276X-9-627

**Published:** 2014-11-23

**Authors:** Sneha Krishnamurthy, Andrea Esterle, Nilesh C Sharma, Shivendra V Sahi

**Affiliations:** 1 Department of Biology, Western Kentucky University, 1906 College Heights Boulevard, Bowling Green 42101-1080, KY, USA

**Keywords:** Spherical AuNPs, Anisotropic Au nanosheets, Photocatalysis, Gradient separation

## Abstract

AuNPs ranging in 20 to 300 nm size were synthesized at a room temperature using *Yucca filamentosa* leaf extract. Diverse nanomaterial morphologies were obtained by varying the extract concentration, reaction pH, and temperature. While low volumes of extract (0.25 and 0.5 mL) induced the formation of microscale Au sheets with edge length greater than 1 μm, high volumes yielded spherical particles ranging from 20 to 200 nm. Varying pH of the solution significantly influenced the particle shape with the production of largely spherical particles at pH 5 to 6 and truncated triangles at pH 2. Separation of multidimensional nanostructures was achieved using a novel method of sucrose density gradient centrifugation. The catalytic function of *Yucca*-derived AuNPs was demonstrated by degradation of a wastewater dye: methylene blue using spectrophotometric measurements over time. Treatment with Au nanosheets and spheres demonstrated methylene blue degradation approximately 100% greater than the activity in control at 60 min.

## Background

Nanoparticles have attracted much attention in recent years due to their unique optoelectronic and physicochemical properties [1]. Their potential applications range from the use in medicine [2], optoelectronic devices [3], sustainable energy systems [4], and food [5] to catalytic functions [6]. A number of green chemical methods employing plant, plant extract, microbes, antioxidants, and ionic liquids have been proposed for the synthesis of environmentally benign metal nanoparticles [7]. One of the simplest methods for gold nanoparticle (AuNP) synthesis is using citrate as a reducing agent for gold salts [8]. However, a major limitation using citrate for synthesis is that AuNPs greater than 50 nm are generally polydispersed in nature. Furthermore, the use of organic solvents and capping agents for controlling the morphology may not always render the synthesized AuNPs as biocompatible and non-toxic [9].

Biological systems, either employing plants or microbes, can be a manufacturing route once pathways are delineated, standardized, and scaled up. The benefit of ‘green chemistry’ can be two-fold. First and foremost, it is an environment-friendly, less intrusive technique with negligible contribution to the generation of hazardous substances. In addition, such biocompatible nanomaterials can be expected to be less reactive to biomolecules, cells, human health, or environment because these products carry surface coatings of biological origin [10,11]. Elemental gold (Au^o^) is considered inert and has been used in food and traditional medicine. However, recent *in vivo* studies highlight potential toxicity of engineered AuNPs [12,13]. It has been reported that the toxicity of (gold) nanoparticles depends on the size, shape, crystal structure, and surface radicles/conjugates.

Recently, various plant products such as biomass of wheat, oat, alfalfa, and leaf extracts of geranium, lemongrass, neem, tamarind, *Aloe vera*, or fruit extract of *Emblica officinalis* were employed for rapid extracellular reduction of ionic Au (III) to Au (0) [14]. *In planta* fabrication of AuNPs has been also reported before [10,15]. Most studies on biomimetic fabrication of nanoparticles were limited by the uncontrolled synthesis of polydisperse nanoparticles and difficulty in separation of particles of various shapes and sizes. Furthermore, physicochemical and optoelectronic properties of the nanomaterial are a strong function of particle shape and size [16]. Especially, non-spherical AuNPs are known for their optical properties due to their ability to exhibit multiple absorption bands. Au nanoprisms, for example, are very promising since they have found applications in cancer therapeutics and biosensing [17,18]. Apart from biomedical applications, AuNPs can be effectively used in applications such as catalysis [11,19] and optical sensing [20]. Therefore, producing biologically derived nanostructures of a desirable size and morphology is critical to their commercial applicability. In light of the above observation, the objectives of this study were to determine the optimal conditions of (i) plant extract concentration, (ii) potassium tetrachloroaurate concentration, (iii) incubation time, (iv) reaction pH, (v) reaction temperature, and (vi) fractionation method for the production of nanomaterial using *Yucca filamentosa* leaf extract. This investigation also demonstrates the catalytic function of a suitable nanostructure using reduction of a wastewater dye.

## Methods

### Preparation of *Y. filamentosa* leaf powder and extract

Extracts from *Hemerocallis fulva* (daylily), *Ilex verticillata* (Hollyberry), *Hedera helix* (English ivy), and *Y. filamentosa* (*Yucca*) were separately prepared and reacted under conditions of a 24-h incubation, 1 mM KAuCl4 concentration, temperature of 37°C, and with no adjustment in pH. Based on variable spectrophotometric measurements, the plant extract showing the highest absorbance, *Y. filamentosa* was chosen for studies in this investigation. (Additional file 1: Figure S1c) *Y. filamentosa* leaves was collected form a local plant nursery, washed to remove any impurities, and dried in an oven for 2 days at 60°C. The leaves were cut into small pieces, pulverized, and sieved using a 20-mesh sieve to get a uniform size range. The fine powder obtained was used for the preparation of *Y. filamentosa* leaf extract (YFLE). Extract stock was prepared by adding 2.5 g of leaf powder to a 500-mL Erlenmeyer flask with 100 mL sterile nanopure water and boiled for 2 min. The boiled mixture was allowed to cool to a room temperature and then filtered using a 0.44-micron millipore sterile filter to remove particulate matter. The same procedure was used for the other plants. Various volumes of the extract (0.25, 0.5, 0.75, 1.0, 1.25, 1.5, and 2.0 mL) were used from the above stock solution. A working volume of 10 mL was maintained for all experiments.

### Biosynthesis of AuNPs

Potassium chloroaurate (KAuCl_4_) was purchased from Sigma-Aldrich, St. Louis, MO, USA. For kinetic studies, 1 mM KAuCl_4_ solution was reacted to 1 mL of YFLE at a room temperature (22°C to 24°C) and pH 4.2 for 6 to 48 h. The following conditions were varied for the optimization of the process of AuNP synthesis: (a) YFLE volumes: 0.25, 0.5, 0.75, 1.0 , 1.25, and 1.5 mL; (b) KAuCl_4_ concentrations: 0.5, 1, 1.5, and 2 mM; (c) pH: pH 1 to pH 6; (d) temperature regime (10°C to 100°C): 10°C, 20°C, 30°C, 40°C, and 100°C.

### Characterization of AuNPs

The presence of synthesized AuNPs was monitored periodically using Perkin Elmer’s LAMBDA 35 UV/vis spectrophotometer (LAMBDA 35 UV–vis spectrophotometer, Perkin Elmer, Waltham, MA, USA). For analysis, 0.1 mL of samples was diluted to 1 mL using nanopure water. The UV–vis spectra were monitored as a function of reaction time, biomaterial dosage, pH, and KAuCl_4_ concentrations. Transmission electron microscope (TEM) images were obtained in order to characterize the size and morphology of the synthesized AuNPs. One part of AuNP stock was diluted with five parts of nanopure water. Five microliters of the diluted AuNP solution was placed on formvar-coated copper grids and allowed to dry over a hot plate. Samples were randomly scanned through using a 120-CX TEM (JEOL JEM, Peabody, MA, USA) at 100 kV. X-ray diffraction was carried out on a Rigaku Miniflex-II (Rigaku, Shibuya-ku, Japan), with a step size of 0.02 using Cu K α radiation.

### Purification and sucrose gradient separation of synthesized biogenic AuNPs

The AuNPs synthesized using plant extract were initially passed though gel filtration column and eluted using distilled water to remove impurities from AuNP colloid. The purified AuNPs were then separated using sucrose gradient centrifugation. A discontinuous sucrose gradient was prepared by making a five-layer step gradient of different concentration of sucrose (10%, 30%, 40%, 50%, 60%, and 70%) in a 2.4-mL polycarbonate ultracentrifuge tube. The tube was added first with 0.5 mL of 70% sucrose, followed by subsequent layering with 0.3 mL of 60%, 50%, 40%, 30%, and 10% sucrose solutions. Typically, 0.2 mL of Au colloid was sonicated in a bucket type sonicator at 80% amplitude with 10 s on-cycle and 10 s off-cycle, for 10 min, and layered on top of the gradient and centrifuged at 500 rpm for 25 min in an ultracentrifuge (SORVALL RC M120EX, Thermo Fisher Scientific, Waltham, MA, USA ). Fractions of the gradient (0.2 mL each) were collected and characterized using TEM following gradient separation.

### Catalytic activity of AuNPs

A heteropolyaromatic dye, methylene blue (MB), was used to evaluate the photocatalytic activity of the various gold nanoparticles. Gold nanoparticles synthesized were diluted to the absorbance of 0.2 at 520 nm using nanopure water. A mixture of stannous chloride and methylene blue was used as a control. Experimental samples contained 3 mL of 1 mM sodium dodecyl sulfate, 10 μL methylene blue, 50 μL stannous chloride, and 100 μL of diluted AuNP solution synthesized under different conditions. Before exposure to illumination, the suspension was stirred in the dark for 10 min to ensure the establishment of adsorption/desorption equilibrium of MB on the sample surfaces. For visible irradiation, direct sunlight was used and all experiments were conducted under similar conditions on the sunny days. Between 12 noon and 1.00 pm (25°C to 27°C), the UV–vis absorption was measured using Perkin Elmer’s LAMBDA 35 UV/vis spectrophotometer (LAMBDA 35 UV/vis spectrophotometer, Perkin Elmer, Waltham, MA, USA). Statistical analysis of photocatalytic activity was analyzed by a one-way analysis of variance (ANOVA) using Microsoft Office Excel 2013. In order to determine the significance, all experimental values were compared to control. Values obtained were the mean of three experiments and were considered significant when *p* <0.05.

## Results and discussion

### Synthesis of AuNPs over reaction time

**Figure 1:**
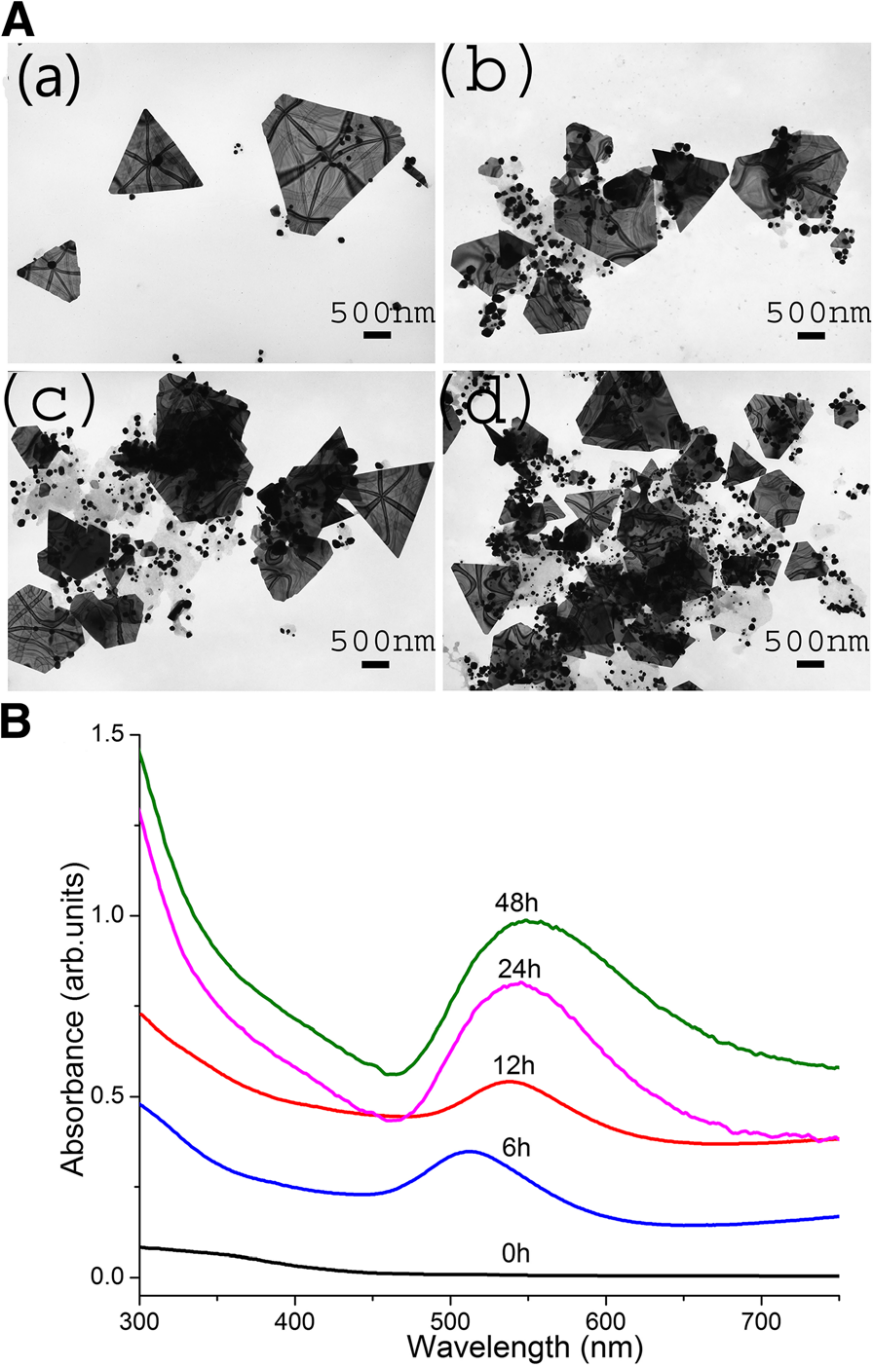
**AuNP synthesis over time. (A)** TEM images showing AuNPs after (a) 6 h, (b) 12 h, (c) 24 h, and (d) 48 h. [Reaction conditions: 1 mL YFLE, 1 mM KAuCl_4_, pH 4.2, and 22°C to 24°C temp]. **(B)** UV–vis spectrometric validation of AuNPs shown in Figure 1A (a-d). The increase in the intensity of absorption peak over time correlates with the temporal increase in AuNPs synthesis.

The addition of 1 mL YFLE to 1 mM KAuCl_4_ (total reaction volume is 10 mL) led to the appearance of ruby red color within 3 h of incubation, indicating the formation of zero valent gold in solution. Gold nanoparticles exhibit vivid colors over time due to surface plasmon resonance phenomenon in the presence of light. Transmission electron microscopic analysis of reaction mixture revealed an increase in the population of nanoparticles with the increase in incubation time of up to 48 h (Figure 1A(a-d)). Nanomaterial shapes included spheres, triangles and cubic sheets in the size range of 10 to 500 nm. Bulk of the nanoparticles was spherical (Additional file 1: Figure S1a). This synthetic pattern was also confirmed with UV–vis spectrophotometric analysis (Figure 1B) of reaction products. Absorption spectra showed a significant increase in the absorption intensity with reaction time, the peak reaching its maxima at 24 h (Figure 1B).

It was observed that the surface plasmon resonance (SPR) band of AuNPS was initially centered at 511 nm and steadily increased as a function of reaction time. A remarkable shift in the peak was noticed as the reaction time advanced from 6 to 24 h. A shift of peak to 545 nm was observed by the end of 24 h, which may be due to an increase in AuNPs size with respect to time. After an incubation period of 24 h, there was no significant increase or shift in SPR peak observed, indicating the complete reduction of Au^3+^ to Au^0^ form [15]. UV-visible spectrophotometry is commonly used to monitor the nanomaterial concentration present in a reaction mixture [21].

**Figure 2:**
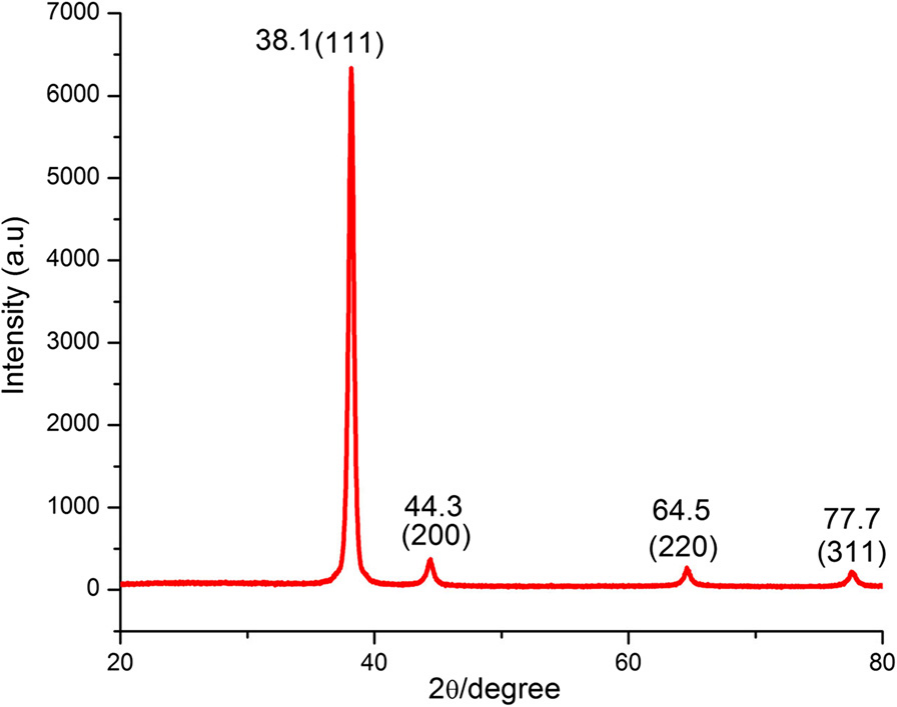
**XRD analysis of gold nanoparticles.** Crystalline nanoparticles represented by four peaks corresponding to standard Bragg reflections (111), (200), (220), and (311) of face centers cubic lattice. The intense peak at 38.1 represents preferential growth in the (111) direction.

The crystallinity of synthesized AuNPs was investigated by an X-ray diffraction (XRD) technique, and corresponding XRD patterns were shown in Figure 2. Gold nanocrystals exhibited four distinct peaks at 2θ = 38.1, 44.3, 64.5, and 77.7. All the four peaks corresponded to standard Bragg reflections (111), (200), (220), and (311) of face center cubic (fcc) lattice. The intense diffraction at 38.1 peak shows that the preferred growth orientation of zero valent gold was fixed in (111) direction. This refers to molecular-sized solids formed with a repeating 3D pattern of atoms or molecules with an equal distance between each part. This XRD pattern is typical of pure Au nanocrystals [22].

### Effects of extract volume and KAuCl_4_ concentration on AuNPs synthesis

**Figure 3:**
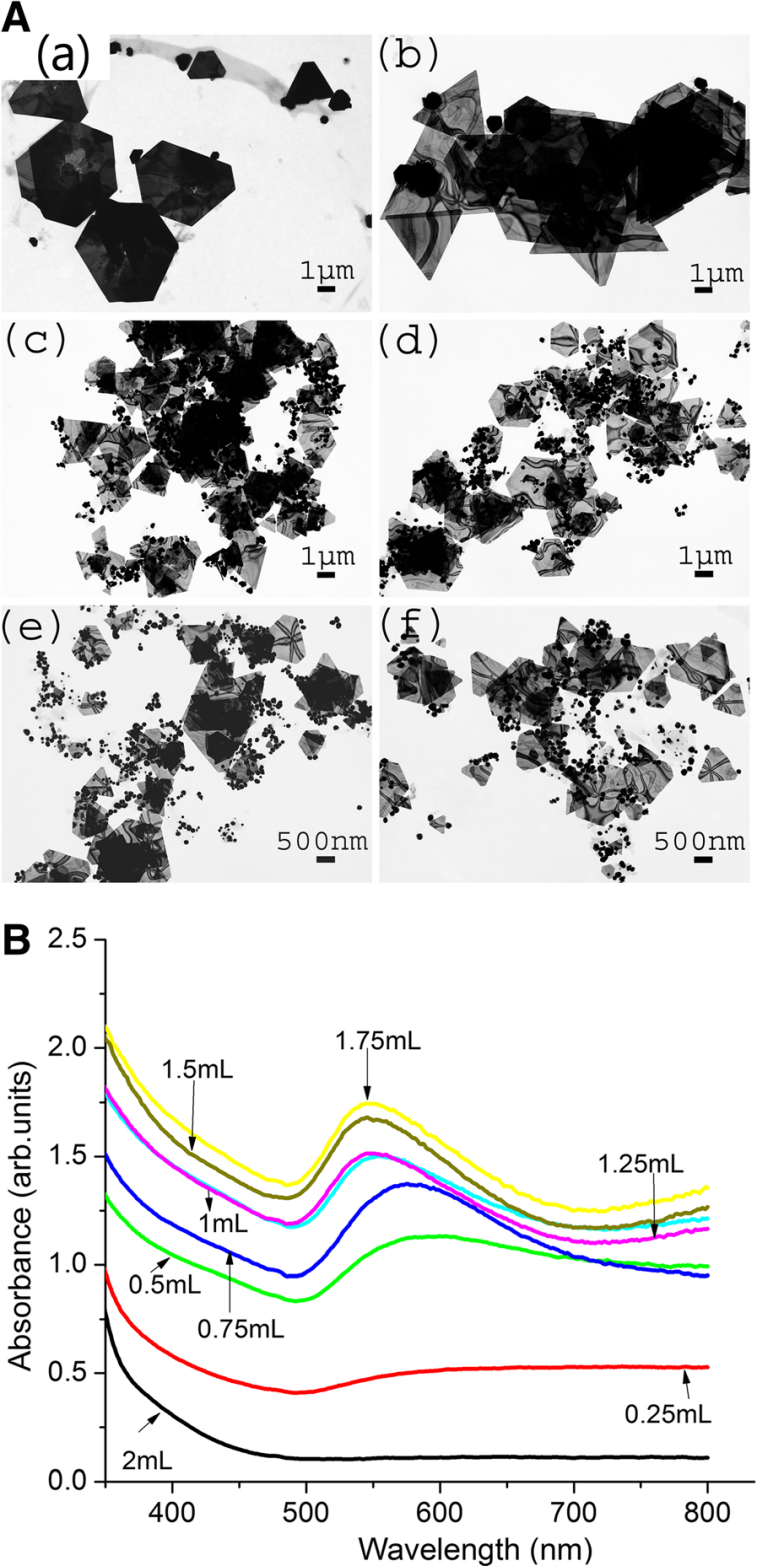
**Effects of different quantities of YFLE on AuNP synthesis. (A)** TEM images of (a) 0.25 mL nanosheets, (b) 0.5 mL nanosheets, (c) 0.75 mL, (d) 1.0 mL polydisperse with spheres and triangles, (e) 1.25 mL polydisperse with spheres and triangles, and (f) 1.5 mL. [Reaction conditions: 1 mM KAuCl_4_, pH 4.2, and 22°C to 24°C temperature]. **(B)** UV–vis spectrometric validation of AuNPs shown in Figure 3A (a-f).

In order to study the effects of YFLE volume on AuNP synthesis, 1 mM KAuCl_4_ was reacted with varying concentrations of extracts (0.25, 0.5, 0.75, 1.0, 1.25, 1.5, and 2.0 mL). Reaction mixtures were incubated for 24 h on an orbital shaker. A preliminary test for the presence of AuNPs was carried out on a UV–vis spectrophotometer. After 24 h of incubation, samples were prepared for characterization using TEM. Figure 3A (a-f) represents multidimensional AuNPs synthesized with various quantities of plant extract. It was evident from the TEM analysis that low concentrations of extract (0.25 and 0.5 mL) induced the formation of a large number of microscale Au sheets with edge length greater than 1 μm (Figure 3A(a-b); Additional file 1: Figure S2). As the volume of extract was increased, a very heterogeneous mixture of particles was obtained. At the volume of 0.25 and 0.5 mL, the particle size ranged from 10 to 3 μm; however, a dramatic decrease in the particle size range (10 to 500 nm) was noticed in the reactions containing 0.75, 1.0, 1.25, or 1.5 mL extract (Figure 3A(c-d); Additional file 1: Figure S2). The spectrophotometric analysis of various reaction mixtures also supported this pattern when the *λ*_max_ value for each extract concentration was observed in the visible range of 540 to 580 nm (Figure 3B). Little or no absorption spectrum was observed at 0.25 or 2 mL concentration (Figure 3B). However, at 0.25 and 2 mL of extract, visual observation showed the settlement of bulk gold in solution within 12 h of reaction (data not shown). Clearly, the absence of absorption peaks at 0.25 and 2 mL extract is due to the formation of bulk gold in the solution. As the volume increased to 1.75 mL, the peak intensity increased with a significant blue shift in SPR peak due to the aggregation of nanoparticles. The increase in peak intensity also reflected an increase in the number of spherical nanoparticles in the solution. The low intensity peak at 0.5 mL extract concentration may be due to the presence of AuNP with larger edge length, and hence, a broader peak [14]. As seen from the TEM images, the gold nanosheets formed at a low concentration may show an absorption in the NIR region rather than the UV–vis region. Similar observations were reported when *A. vera* extract was used to synthesize gold and silver nanoparticles [23]. At a low concentration, few molecules of a reducing agent are available to cap the synthesized AuNPs, which lead to the destabilization and aggregation of the AuNPs due to Oswald ripening [24]. A higher reducing agent concentration stabilizes the particles. However, concentrations of extract higher than 1.75 mL also demonstrated the aggregation of gold ion to its bulk form. It can also be noted that visual observations on 0.25 and 2 mL extract concentrations indicated the settlement of bulk gold in solution within 12 h of reaction (data not shown). Clearly, the absence of absorption peaks at 0.25 and 2 mL extract concentration is due to the formation of bulk gold in solution. Hence, the reducing agent concentration plays a crucial role in the stability of AuNPs. Other studies [24,25] have also indicated a similar finding. While various studies demonstrate the synthesis of metal nanoparticles using plant extracts, the mechanism involving reduction is still unsettled. One of the earliest studies on mechanism of nanoparticles synthesis using geranium extracts have the reported role of water soluble proteins and secondary metabolites in metal ion reduction [26]. Other reports implicated the role of water soluble compounds like flavonoids, terpenoids, and thiamine as capping agent for nanoparticle synthesis. An investigation designed to study the mechanism of Au and Ag ion reduction using FTIR and cyclic voltammetry revealed a post-reduction decrease in polyphenols/flavonoids content in tea extract, leading to the conclusion that polyphenols or flavonoids are the main reducing agents involved in the process of nanometal synthesis [27]. Any nanoparticle formation involves the following: (a) nucleation, (b) crystal growth of the particle, and (c) secondary reduction on the surface to stop crystal growth. However, nanoparticle synthesis and crystal growth are affected by many factors including pH, temperature, and reductant concentration [14,27].

**Figure 4:**
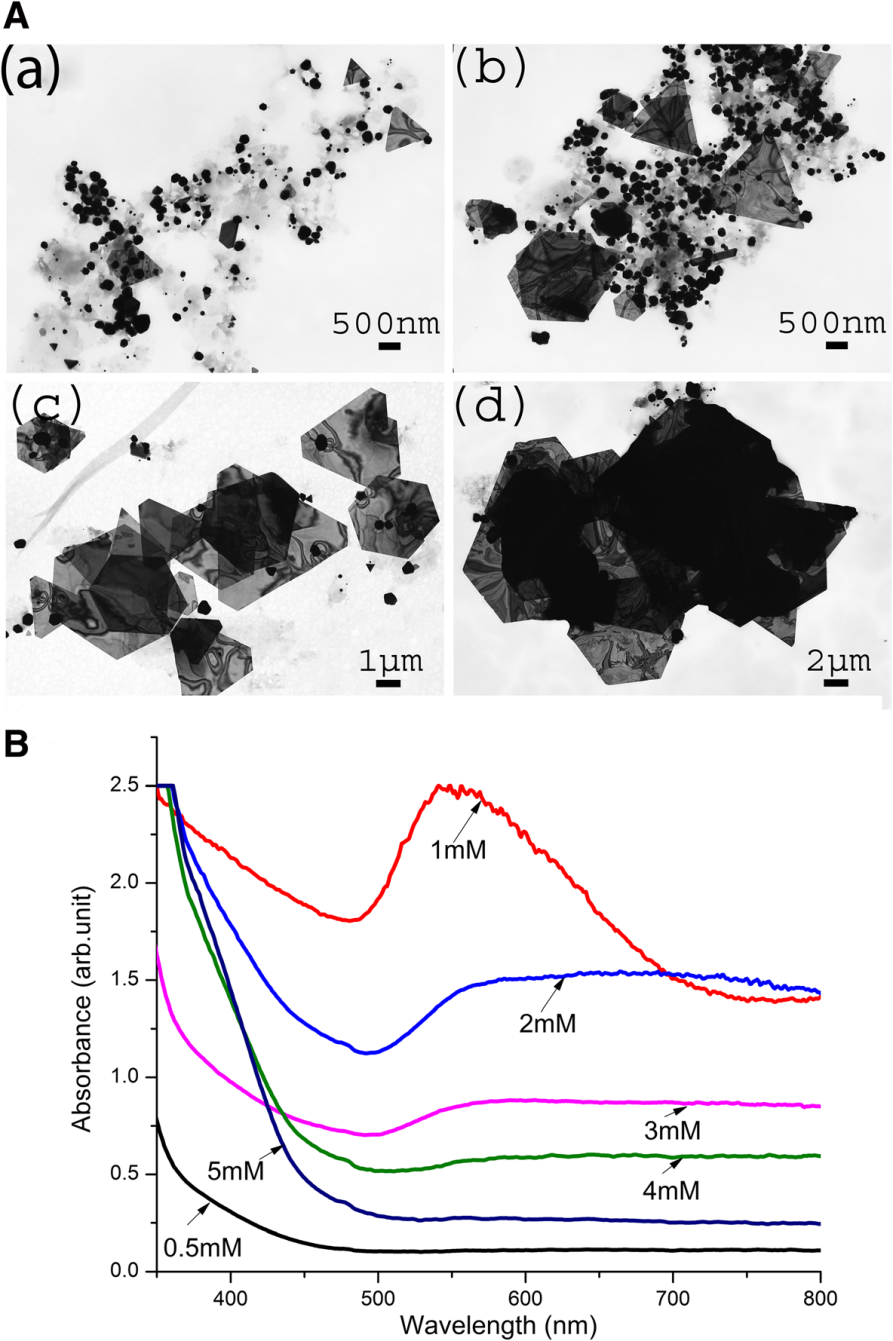
**Effects of KAuCl**_**4 **_**concentrations on AuNP synthesis. (A)** TEM images at (a) 0.5 mM KAuCl_4_, abundance of spheres; (b) 1 mM KAuCl_4_, abundance of spheres; (c) 1.5 mM KAuCl_4_, formation tending to bulk gold; and (d) 2 mM KAuCl_4_, bulk gold [Reaction conditions: 1 mL YFLE, pH 4.2, and 22°C to 24°C temperature]. **(B)** UV–vis spectrophotometry validation of AuNPs shown in Figure 4A (a-d).

In order to understand the effect of KAuCl_4_ concentration on the formation of AuNPs, 1 mL of plant extract was reacted with varying concentrations of KAuCl_4_ (0.5, 1, 1.5, 2, 3, 4, and 5 mM). Based upon the above observations, 1 mL extract was chosen as the optimum concentration of reducing agent for the reaction mixture. TEM micrographs (Figure 4A(a-f)) show the presence of spherical nanoparticles at lower concentrations of KAuCl_4_ solution (0.5, 1 mM) while microscale gold sheets at higher concentrations (1.5 and 2 mM) (Additional file 1: Figure S3) [28]. It is interesting to observe that Au sheets formed at 1.5 and 2 mM of gold salt possess an average particle size of c.a 2 and 4 μm, respectively (Additional file 1: Figure S3). Figure 4B presents the absorption spectra of AuNPs synthesized with various concentrations of gold salt. It is evident from the above observations that 1 mM was the optimum concentration of gold salt for synthesis of AuNPs. A small SPR band was obtained at 2 and 3 mM gold salt concentration. No peak for AuNPs was observed at 4 and 5 mM gold salt. High metal salt concentration led to a high reaction rate thereby increasing the nucleation process, which triggers the formation of more nuclei [24,28]. This subsequently induced the formation of larger particles with broader size distribution. Furthermore, AuNPs synthesized at high concentrations are known to be unstable due to the colloidal instability at high ionic strength [25,29]. Though the optimal concentration of 1 mM produced the highest peak, nanomaterials were polydispersed with the presence of spherical, hexagonal, triangular, and rod-shaped particles. It is noteworthy that the concentration of reactant and reductant exerts a strong effect on the morphology of nanoparticles [23-25]. This can be used as an effective tool in controlling the optical properties of biogenic nanoparticles. Shankar et al. [30] were the first group to report the synthesis of Au nanoprisms using lemon grass extract, by controlling the extract concentration for reduction of Au^3+^ to its zero valent form. The role of aldehyde and ketones was implicated in the formation of microscale Au sheets with metallic sheen; however, little is known about the interaction of aldehyde/ketones with AuNPs [30]. Aldehyde and ketones are also suspected to direct the crystal growth of nanoparticles. Although chemical composition of *Y. filamentosa* has not been fully characterized, the floral head space was recently reported to contain abundant amounts of saponins, mainly consisting of homoterpenes [31]. Saponins are natural glycosides of steroids or polycyclic triterpenes that might be involved in the reduction of Au ions and stabilization of AuNPs.

### Effects of pH and temperature on AuNP biosynthesis using YFLE

AuNP synthesis by *Yucca* extract was carried out under a range of pH conditions (pH 1 to 6) (Additional file 1: Figure S4). TEM analysis demonstrated the efficient formation of AuNPs at acidic pH (pH 3 to 5) (Additional file 1: Figure S4A (c-e)). It is evident from Additional file 1: Figure S4A (a-f) that decreasing pH of the solution to pH 2 induced an aggregation of AuNPs with as much as 40% of truncated triangles (Additional file 1: Figure S5). No nanoparticle formation was observed at pH 1 (Additional file 1: Figure S4A). This is probably due to the leaching of gold and denaturation of the biomolecules in a highly acidic condition [22]. The nanoparticles formed at acidic pH (pH 2 and 3) were polydisperse (Additional file 1: Figure S4A (b-c)). It is interesting to note that the nanoparticle size decreased with an increase in pH of the solution. Starnes et al. (10) and Sneha et al. (22) observed a similar pattern. Reaction mixtures at a pH 5 to 6 led to the formation of nanoparticles in the range of 10 to 100 nm (Additional file 1: Figure S4A e-f). Also increase in pH directly correlated with the formation of spherical particles, almost reaching monodispersity at pH 6. Truncated triangles UV–vis spectral peaks also confirmed the pattern observed above (Additional file 1: Figure S4B). The SPR peak significantly dropped at pH 6, indicating a poor yield of AuNPs. Findings in the present study are in agreement with another report [32] wherein pH 3 to 5 was found to be an optimal range. Low pH leads to protonation and neutralization of carboxyl groups in the extract promoting the interaction between the AuCl^4-^ ion and the biomolecule. However, the nanoparticles continue to grow until reductant and biomolecule are available. Thus, the enhanced interaction of AuCl^4-^ ions with biomolecules in acidic pH also contributes to the growth of crystals [22,32]. This explains the polydispersity in the size of nanoparticles at this range of pH (3 to 5). No studies were possible beyond pH 6 due to the precipitation of gold salt in the solution.

In order to control size of nanoparticle, the effect of different temperatures was scrutinized on AuNP synthesis. TEM analysis revealed the change in the size and shape of the synthesized AuNPs at different temperatures (Additional file 1: Figure S6 (a-d)). It is evident from TEM images that the nanoparticles synthesized at 10°C were flocculated and aggregated, the size exceeding a micrometer (Additional file 1: Figure S7). As the reaction temperature reached 20°C, the shape of nanoparticles became mainly anisotropic in nature (Additional file 1: Figure S6b). AuNPs synthesized at higher temperatures were polydisperse (Additional file 1: Figure S6 (c-d). The nanoparticle reduction was rapid at 100°C and the process was complete with few minutes of reaction. The majority of AuNPs synthesized at this temperature were in the size range of 20 to 40 nm, and the particles synthesized were mainly spherical or elliptical in shape along with some tiny triangular nanoparticles (Additional file 1: Figure S6d). This pattern of nanoparticle growth at low temperature, obtained in this study, is reasonable, since nucleation and growth is a kinetically driven process. Hence, the rate of reaction slows down at a low temperature which allows nucleation and crystal growth at a slow rate. Whereas, at high temperature, due to a rapid rate of reaction, the primary nucleation is immediately followed by a secondary reduction on the primary nuclei; hence, particle growth is stalled. Furthermore, the high rate of reaction also leads to the fast consumption of available ions, reducing them to zero valent form [22,28].

### Separation of biogenic AuNPs by sucrose density gradient centrifugation

**Figure 5:**
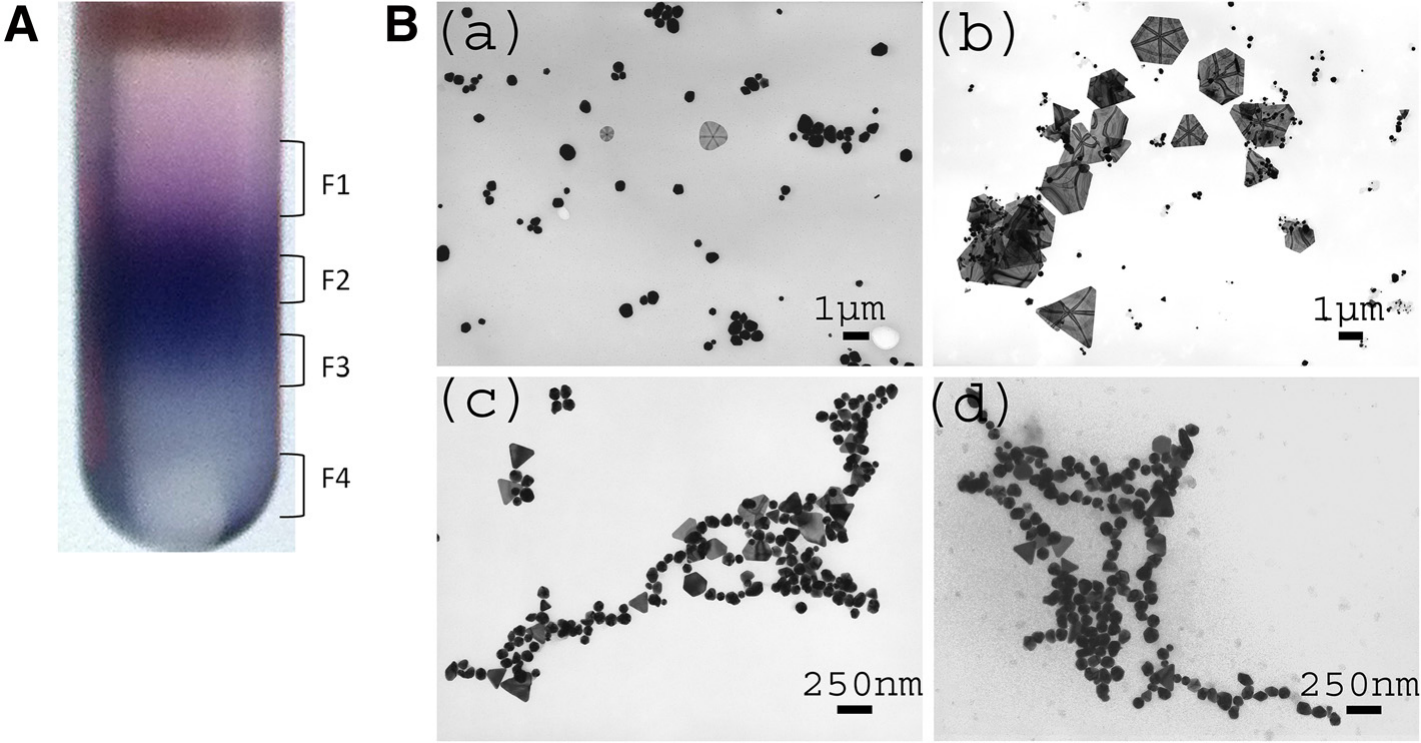
**Fractionation and separation of AuNPs. (A)** Fractionation of AuNPs using sucrose density gradient centrifugation: F1-F4. **(B)** Separation of mostly spherical particles (a) from column fraction F1, large nanosheets (b) from fraction F2, and nanospheres: 10 to 30 nm (c, d) from fractions F3.

The nanoparticles were separated based on the size and shape by density gradient centrifugation of 10% to 70% sucrose (Figure 5A,B). TEM micrograph (Figure 5B (a-d)) shows the presence of spherical nanoparticles separated from 30% fraction (Figure 5A-F1 fraction). This fraction dominated in monodisperse AuNPs with little aggregates. The spherical AuNPs separated from F1 band measured about 80 nm in diameter. Interestingly, F2 had polydisperse collection containing various shapes and sizes. The average size of large nanoparticles obtained from this fraction was 1.5 μm (Figure 5B(b)). The nanoparticles obtained from F3 fraction (Figure 5A) were mostly spherical in nature (Figure 5B(c-d)). The AuNPs separated from this fraction were significantly smaller than those of fractions F1 and F2. Another feature of F3 particles was the chain-like growth of smaller particles. Obviously, fraction F3 had a high concentration sucrose layer that might have contributed to the chain-like growth. A similar phenomenon was reported wherein Zirconia nanochains were synthesized using *Curcuma longa* tuber extract [33]. Again, fraction F4 had smaller particles in the range of 10 to 40 nm while forming large aggregates measuring in micrometers. Larger aggregates might have formed in the solution due to the Oswald ripening in presence of reducing agent and reductant [34]. This study provides an easy and efficient method of separation of biogenic nanoparticles based on size and shape effects.

### Catalytic activity of AuNPs

**Figure 6:**
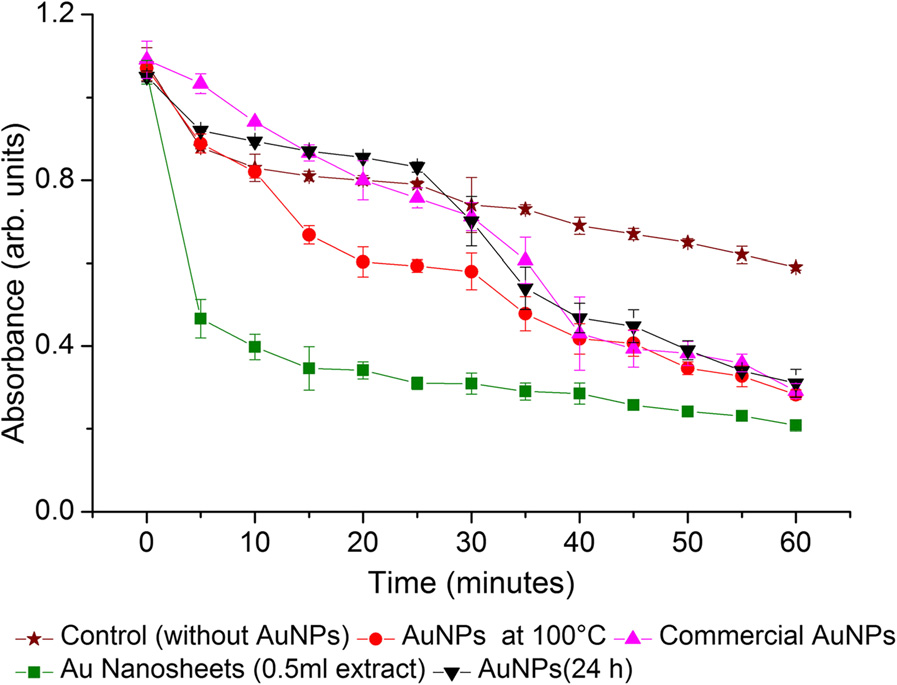
Degradation of methylene blue by different nanostructures over 0 to 60 min.

Photocatalysis of dyes and other pollutants is an effective remediation strategy for waste water treatment. In this study, the catalytic activity of AuNPs was substantiated by carrying out the degradation of MB dye using SnCl_2_ and biogenic AuNPs. Nanoparticles of different shapes and sizes obtained under above physicochemical conditions were tested for their catalytic properties. They included the following: (i) anisotropic microscale sheets obtained using 0.5 mL extract, (ii) spherical nanoparticles obtained at 100°C, (iii) commercially obtained citrate capped AuNPs for comparison, and (iv) polydisperse AuNPs synthesized at room temperature and native pH 4.2 of gold salt using 1 mL *Yucca* extract. The decolorization of MB using SnCl_2_ was monitored by a spectrophotometer (Figure 6). Addition of different AuNPs in MB solution resulted in reduction reactions over a period of time. In this case, MB with SnCl_2_ was used as control. It is evident from Figure 6 that AuNPs enhanced significantly the degradation of MB dye. This increase in photocatalytic activity in the presence of AuNPs is due to its ability to act as a sink for photoinduced electrons and promote the charge transfer process for catalytic activity in presence of SnCl_2_[35]. It can be clearly seen from the above experiment that the catalytic activity in presence of microscale gold sheets was most efficient for the degradation of dye (Figure 6). It is interesting to observe that the absorbance peak falls drastically in the first 10 min of reaction and then gradually decreases over a period of time. The AuNPs synthesized at 100°C and the heterogeneous mixture of AuNPs show an activity similar to those of commercially obtained citrate capped AuNPs. In the absence of AuNPs (control), only 46% of the dye was found degraded after 60 min of reaction. On the contrary, the photocatalytic degradation of the dye was found to be 89% and 81% in presence of Au nanosheets and spherical AuNPs, respectively. (Additional file 1: Table S1) The results clearly indicate that AuNPs enhance the photocatalytic degradation of methylene blue in the presence of SnCl_2_. Single factor ANOVA was performed in order to analyze the variance in different samples. The *p* value for this analysis was found to be *p* <0.05, which is an evidence for significant difference in photocatalytic activity of different samples. Anisotropic particles possess better positional order of FCC lattice in comparison with the spherical counterparts [36]. This allows face-to-face interaction and immediate absorption of MB on microscale Au sheets [35], due to which we can see an immediate drop in absorbance during the initial stage of the reaction. Hence, anisotropic AuNPs degrade MB dye more efficiently. These results clearly indicate that properties of biogenic nanoparticles can be controlled using various parameters and made suitable for use as an efficient photocatalyst.

## Conclusions

We have, for the first time, demonstrated the difference in photocatalytic activity of size-dependent biogenic nanoparticles synthesized under different physicochemical conditions. Microscale anisotropic Au sheets synthesized using 0.5 mL plant extract exhibited better photocatalytic activity than their spherical counter parts. Although a number of reports show the synthesis of biogenic metal nanoparticles, few reports present the practical application of nanoparticles synthesized through biological means. Furthermore, the ease of separation of AuNPs using a sucrose gradient centrifugation is an improvement to research using biological methods for nanoparticle fabrication.

## Competing interests

The authors declare that they have no competing interests.

## Authors’ contributions

SK, AE, and SS proposed the idea; SK designed the experiments. SK and AE performed the experiments. SK, NS, and SS wrote and revised the paper. All authors read and approved the final manuscript.

## Supplementary Material

Additional file 1: **Illustrations showing various processes of AuNP synthesis.** Figures depicting gold nanoparticle synthesis over a period of time **(Figure S1)**, dependence of nanoparticle dimension on different plant extract concentrations **(Figure S2)**, gold salt concentrations **(Figure S3)**, reaction mixture pH **(Figures S4 and 5)**, reaction temperature **(Figures S6 and S7)**, and extent of methylene blue degradation **(Table S1)**. Click here for file

## References

[B1] Chen HM Liu R-S Architecture of metallic nanostructures: synthesis strategy and specific applications J Phys Chem C 2011 115 3513 3527 10.1021/jp108403r

[B2] Mendes PM Cellular nanotechnology: making biological interfaces smarter Chem Soc Rev 2013 42 9207 9218 10.1039/c3cs60198f 24097313 PMC3984013

[B3] Suryanarayanan R Rao MSR, Okada T Zinc oxide: from optoelectronics to biomaterial—a short review ZnO Nanocrystals and Allied Materials, Volume 180 2014 India: Springer 289 307

[B4] Vilatela JJ Eder D Nanocarbon composites and hybrids in sustainability: a review Chem Sus Chem 2012 5 456 478 10.1002/cssc.201100536 22389320

[B5] Sozer N Kokini JL Nanotechnology and its applications in the food sector Trends Biotechnol 2009 27 82 89 10.1016/j.tibtech.2008.10.010 19135747

[B6] Dhakshinamoorthy A Garcia H Catalysis by metal nanoparticles embedded on metal-organic frameworks Chem Soc Rev 2012 41 5262 5284 10.1039/c2cs35047e 22695806

[B7] Hebbalalu D Lalley J Nadagouda MN Varma RS Greener techniques for the synthesis of silver nanoparticles using plant extracts, enzymes, bacteria, biodegradable polymers, and microwaves ACS Sustainable Chem Eng 2013 1 703 712

[B8] Ftouni J Penhoat M Addad A Payen E Rolando C Girardon J-S Highly controlled synthesis of nanometric gold particles by citrate reduction using the short mixing, heating and quenching times achievable in a microfluidic device Nanoscale 2012 4 4450 4454 10.1039/c2nr11666a 22722332

[B9] Thakkar KN Mhatre SS Parikh RY Biological synthesis of metallic nanoparticles Nanomed Nanotechnol 2010 6 257 262 10.1016/j.nano.2009.07.002 19616126

[B10] Starnes DL Jain A Sahi SV In planta engineering of gold nanoparticles of desirable geometries by modulating growth conditions: an environment-friendly approach Environ Sci Technol 2010 44 7110 7115 10.1021/es101136q 20698550

[B11] Sharma NC Sahi SV Nath S Parsons JG Gardea-Torresdey JL Pal T Synthesis of plant-mediated gold nanoparticles and catalytic role of biomatrix-embedded nanomaterials Environ Sci Technol 2007 41 5137 5142 10.1021/es062929a 17711235 PMC2518977

[B12] Zhang XD Wu D Shen X Liu PX Fan FY Fan SJ In vivo renal clearance, biodistribution, toxicity of gold nanoclusters Biomaterials 2012 33 4628 4638 10.1016/j.biomaterials.2012.03.020 22459191

[B13] Lasagna-Reeves C Gonzalez-Romero D Barria MA Olmedo I Clos A Ramanujam VMS Urayama A Vergara L Kogan MJ Soto C Bioaccumulation and toxicity of gold nanoparticles after repeated administration in mice Biochem Biophys Res Commun 2010 393 649 655 10.1016/j.bbrc.2010.02.046 20153731

[B14] Mittal AK Chisti Y Banerjee UC Synthesis of metallic nanoparticles using plant extracts Biotechnol Adv 2013 31 346 356 10.1016/j.biotechadv.2013.01.003 23318667

[B15] Sharma V Park K Srinivasarao M Colloidal dispersion of gold nanorods: historical background, optical properties, seed-mediated synthesis, shape separation and self-assembly Mat Sci Eng R 2009 65 1 38 10.1016/j.mser.2009.02.002

[B16] Kumar SK Krishnamoorti R Nanocomposites: structure, phase behavior, and properties Annu Rev Chemi Biomol Eng 2010 1 37 58 10.1146/annurev-chembioeng-073009-100856 22432572

[B17] Bao C Beziere N del Pino P Pelaz B Estrada G Tian F Ntziachristos V de la Fuente JM Cui D Nanoprisms: gold nanoprisms as optoacoustic signal nanoamplifiers for in vivo bioimaging of gastrointestinal cancers (small 1/2013) Small 2013 9 67 67 10.1002/smll.201370007 23001862

[B18] Bao C Beziere N del Pino P Pelaz B Estrada G Tian F Ntziachristos V de la Fuente JM Cui D Gold nanoprisms as optoacoustic signal nanoamplifiers for in vivo bioimaging of gastrointestinal cancers Small 2013 9 68 74 10.1002/smll.201201779 23001862

[B19] Stratakis M Garcia H Catalysis by supported gold nanoparticles: beyond aerobic oxidative processes Chem Rev 2012 112 4469 4506 10.1021/cr3000785 22690711

[B20] Guffey MJ Scherer NF All-optical patterning of Au nanoparticles on surfaces using optical traps Nano Lett 2010 10 4302 4308 10.1021/nl904167t 20925400

[B21] Haiss W Thanh NT Aveyard J Fernig DG Determination of size and concentration of gold nanoparticles from UV–vis spectra Anal Chem 2007 79 4215 4221 10.1021/ac0702084 17458937

[B22] Sneha K Sathishkumar M Kim S Yun YS Counter ions and temperature incorporated tailoring of biogenic gold nanoparticles Process Biochem 2010 45 1450 1458 10.1016/j.procbio.2010.05.019

[B23] Chandran SP Chaudhary M Pasricha R Ahmad A Sastry M Synthesis of gold nanotriangles and silver nanoparticles using Aloe vera plant extract Biotechnol Progr 2006 22 577 583 10.1021/bp0501423 16599579

[B24] Kimling J Maier M Okenve B Kotaidis V Ballot H Plech A Turkevich method for gold nanoparticle synthesis revisited J Phys Chem B 2006 110 15700 15707 10.1021/jp061667w 16898714

[B25] Badwaik VD Bartonojo JJ Evans JW Sahi SV Willis CB Dakshinamurthy R Single-step biofriendly synthesis of surface modifiable, near-spherical gold nanoparticles for applications in biological detection and catalysis Langmuir 2011 27 5549 5554 10.1021/la105041d 21480600

[B26] Shankar SS Ahmad A Pasricha R Sastry M Bioreduction of chloroaurate ions by geranium leaves and its endophytic fungus yields gold nanoparticles of different shapes J Mat Chem 2003 13 1822 1826 10.1039/b303808b

[B27] Akhtar MS Panwar J Yun Y-S Biogenic synthesis of metallic nanoparticles by plant extracts ACS Sustainable Chem Eng 2013 1 591 602 10.1021/sc300118u

[B28] Dwivedi AD Gopal K Biosynthesis of silver and gold nanoparticles using *Chenopodium album* leaf extract Colloids Surf A 2010 369 27 33 10.1016/j.colsurfa.2010.07.020

[B29] Zabetakis K Ghann WE Kumar S Daniel MC Effect of high gold salt concentrations on the size and polydispersity of gold nanoparticles prepared by an extended Turkevich-Frens method Gold Bull 2012 45 203 211 10.1007/s13404-012-0069-2

[B30] Shankar SS Rai A Ankamwar B Singh A Ahmad A Sastry M Biological synthesis of triangular gold nanoprisms Nat Mater 2004 3 482 488 10.1038/nmat1152 15208703

[B31] Svensson GP Hickman MO Jr Bartram S Boland W Pellmyr O Raguso RA Chemistry and geographic variation of floral scent in *Yucca filamentosa* (Agavaceae) Am J Bot 2005 92 1624 1631 10.3732/ajb.92.10.1624 21646079

[B32] Gardea-Torresdey JL Tiemann KJ Gamez G Dokken K Cano-Aguilera I Furenlid LR Renner MW Reduction and accumulation of gold (III) by *Medicago sativa* alfalfa biomass: X-ray absorption spectroscopy, pH, and temperature dependence Environ Sci Technol 2000 34 4392 4396 10.1021/es991325m PMC4666683 26635419

[B33] Sathishkumar M Sneha K Yun YS Green fabrication of zirconia nano-chains using novel *Curcuma longa* tuber extract Mater Lett 2013 98 242 245

[B34] DongáKim W YoungáWoo J Metal tips on pyramid-shaped PbSe/CdSe/CdS heterostructure nanocrystal photocatalysts: study of Ostwald ripening and core/shell formation Chem Commun (Camb) 2014 50 1719 1721 10.1039/c3cc48919a 24395043

[B35] Lee J Shim HS Lee M Song JK Lee D Size-controlled electron transfer and photocatalytic activity of ZnO-Au nanoparticle composites J Phys Chem Lett 2011 2 2840 2845 10.1021/jz2013352

[B36] Jones MR Macfarlane RJ Lee B Zhang JA Young KL Senesi AJ Mirkin CA DNA-nanoparticle superlattices formed from anisotropic building blocks Nat Mater 2010 9 913 917 10.1038/nmat2870 20890281

