# Supplementary material for: Yucca-derived synthesis of gold nanomaterial and their catalytic potential
Source: Nanoscale Res Lett. 2014 Nov 23;9(1):627. doi: 10.1186/1556-276X-9-627 (PMC4256969; doi:10.1186/1556-276X-9-627)
Supplement: Additional file 1: — Illustrations showing various processes of AuNP synthesis. Figures depicting gold nanoparticle synthesis over a period of time (Figure S1), dependence of nanoparticle dimension on different plant extract concentrations (Figure S2), gold salt concentrations (Figure S3), reaction mixture pH (Figures S4 and 5), reaction temperature (Figures S6 and S7), and extent of methylene blue degradation (Table S1). [file 1556-276X-9-627-S1.docx]

Figure S1: Reaction kinetics of gold nanoparticle synthesis using YFLE. (a) Temporal studies showed the presence of mainly triangular particle in the initial stages of reaction. As the reaction progressed, the number of spherical particles and other anisotropic nanoparticle increased in number. The percentage of spherical nanoparticle remained consistent throughout the process. However, the percentage of hexagonal sheets increased as the reaction progressed. (b) The number of particles observed increased as the reaction time progressed. The numbers of particles were calculated by observing the total particle count in an area of 48 sq µm for each sample. The particle count appeared to be constant after 12h of reaction. (c) [*Hemerocallisfulva*](http://en.wikipedia.org/wiki/Hemerocallis_fulva)(Day lily),*Ilex verticillata* (Holly Berry), *Hedera helix* (English Ivy), *Yuca filamentosa* (Yucca) were reacted under conditions of a 24 hour time period, a 1mM KAuCl4 concentration, a temperature of 37^o^C, and no adjustment in pH. Spectrophotometer measurements were made variables and the plant having the highest absorbance, *Yucca filamentosa*, was chosen to be worked with.

| a) |  |
| --- | --- |
| b) |  |
| c) | 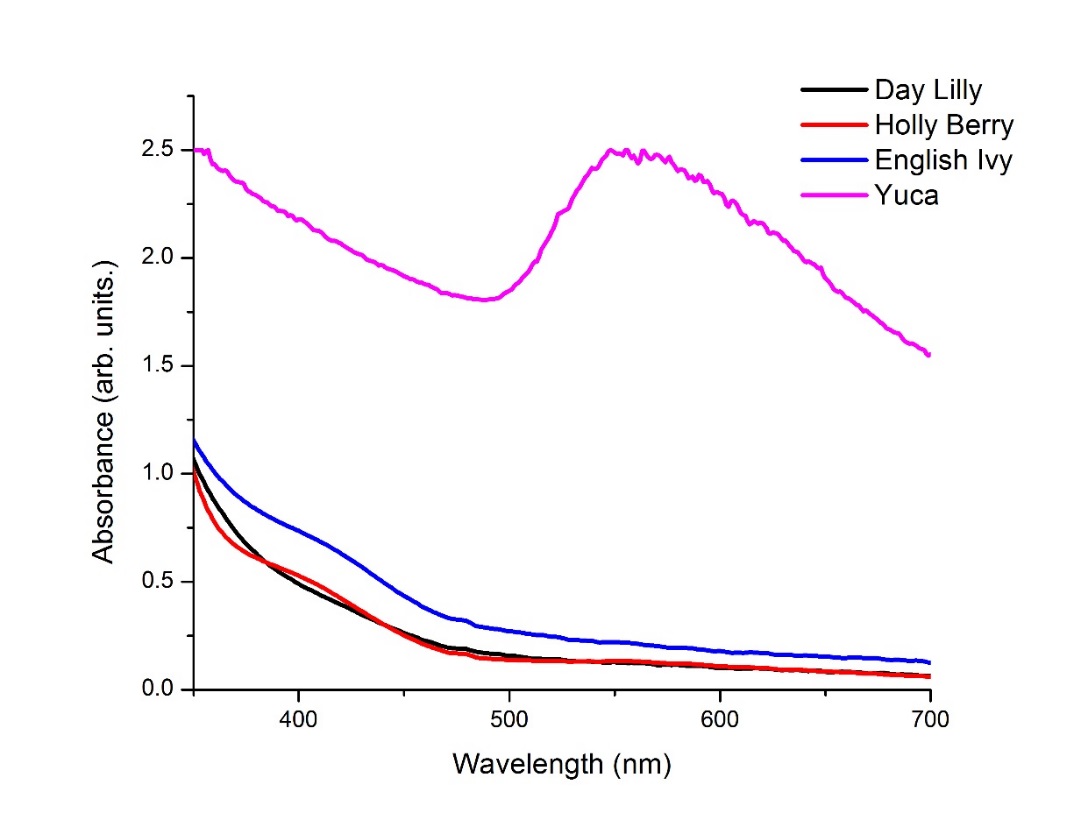 |

Figure S2: Particle size distribution at different extract concentrations.

|  |  |
| --- | --- |
|  |  |
|  |  |

Figure S3: Particle size distribution at different concentrations of gold

|  |  |
| --- | --- |
|  |  |

**Figure S4A (a-f):** Effects of pH on the fabrication of AuNPs: TEM image (a) pH 1, (b) pH 2, (c) pH 3, (d) pH 4, (e) pH 5, and (f) pH 6 [Reaction conditions: 1mM KAuCl_4_, 1mL YFLE and 22-24^o^C temp] Fig. 4B: UV-vis spectrometric validation of AuNPs shown in Fig. 4A (a-f). pH 4 was found to be optimum for nanoparticles synthesis. Sufficient absorbance was observed at pH 3 and pH 5 indicating the formation of nanoparticles with good optical properties.

| A | 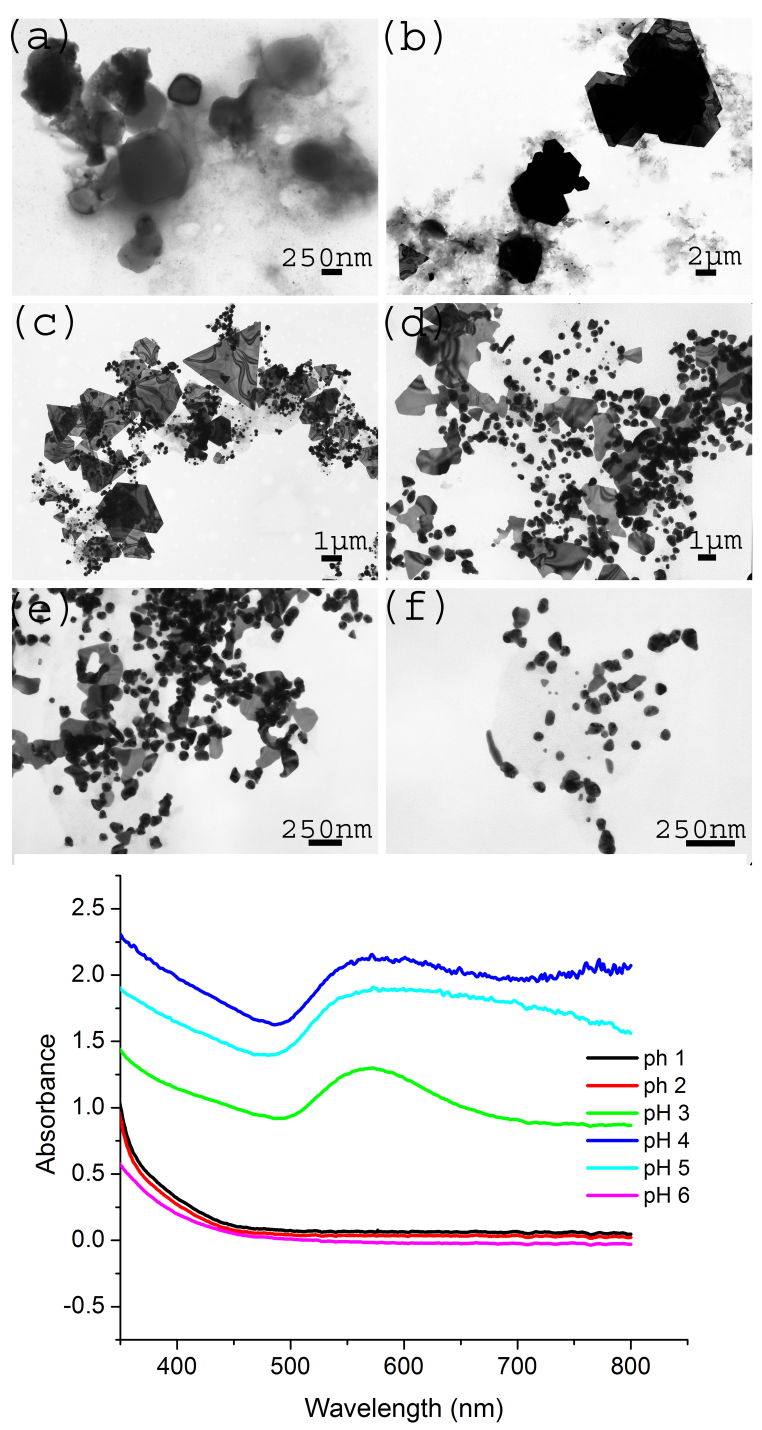 |
| --- | --- |
| B |  |

**Figure S5**: Particle distribution on the basis of morphology at different pH of the reaction mixture

**Figure S6:** Effects of temperature on the fabrication of AuNPs: TEM image (a) 10°C: anisotropic sheets, (b) 20°C: abundance of anisotropic sheets, (c) 40°C, and (d) 100°C: abundance of spheres [Reaction conditions: 1mM KAuCl_4_, 1mL YFLE and pH 4.2]


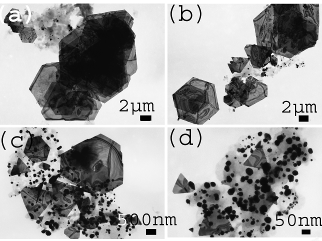


**Figure S7**: Average particle size of nanoparticles synthesized at various temperature. Lower temperatures assisted the synthesis of larger nanoparticles mostly consisting of anisotropic nanosheets.

**Table S1:**

| **Extent of methylene blue degradation after 60 min of reaction (%)** | | | | | |
| --- | --- | --- | --- | --- | --- |
|  | Control | Nanosheet | Spherical | Commercial AuNPs | Mixed AuNPs |
| Replicate 1 | 42.58415842 | 86.37735849 | 80.64485981 | 82.30275229 | 75.47619048 |
| Replicate 2 | 42.80079051 | 86.8728558 | 82.5396648 | 90.37288136 | 66.69512195 |
| Replicate 3 | 43.82997033 | 93.79279279 | 82.14558824 | 84.85840708 | 73.19417476 |
